# Mid-Term Migration Pattern of a Cemented Collared Anatomical Stem—A Retrospective Study Using EBRA-FCA

**DOI:** 10.3390/jcm13175187

**Published:** 2024-09-01

**Authors:** Philipp Blum, Johannes Neugebauer, Alexander Keiler, David Putzer, Julius Watrinet, Stephan Regenbogen, Dietmar Dammerer

**Affiliations:** 1 Department of Trauma Surgery, BG Trauma Center Murnau, 82418 Murnau, Germany; 2 Department of Orthopaedics and Traumatology, Krems University Hospital, 3500 Krems, Austria; 3 Karl Landsteiner Private University for Health Sciences, 3500 Krems, Austria; 4 Department of Orthopaedics and Traumatology, Medical University of Innsbruck, 6020 Innsbruck, Austria; 5 Department of Experimental Orthopaedics, Medical University of Innsbruck, 6020 Innsbruck, Austria; 6 Department of Orthopaedic Sports Medicine, Technical University of Munich, 81675 Munich, Germany; 7 Department of Traumatology, BG Trauma Center Ludwigshafen, University of Heidelberg, 67071 Ludwigshafen, Germany

**Keywords:** total hip arthroplasty, Einzel-Bild-Röntgen-Analyse, cemented, subsidence

## Abstract

**Background**: Aseptic loosening is one of the leading causes of stem revision. Einzel Bild Röntgen Analyse–Femoral Component Analysis allows for the detection of distal stem migration, which is used as a predictive factor for implant longevity. This study aims to demonstrate the migration behavior of a cemented collared anatomical stem. **Methods**: This study retrospectively examined all patients who received a cemented Lubinus SP II stem (Waldemar Link, Hamburg, Germany) between 2003 and 2019. We used the EBRA-FCA software (University of Innsbruck, Austria) to determine the migration patterns and thoroughly examined the patients’ medical histories. In addition, the potential influence of femoral configuration and BMI on the migration behavior was assessed. **Results**: This study included 61 patients (48 females and 13 males) with a total of 61 stems that met our inclusion criteria. The mean age at surgery was 76 years (ranging from 30 to 93 years). According to EBRA-FCA migration analysis, a median subsidence of 0.7 mm was observed at 24 months and at the final follow-up (median 78 months). Distal stem migration was significantly higher at the 6-month time point in patients with Dorr type A femurs compared to Dorr type B femurs (*p* = 0.016). Body mass index (BMI) had no significant effect on stem migration. **Conclusions**: The measured subsidence of the Lubinus SP 2 stem using EBRA-FCA was below established thresholds, indicating excellent long-term outcomes. Although there was significantly increased subsidence in Dorr type A femurs during the initial 6 months, thereafter, no statistically significant difference was observed compared to Dorr type B femurs.

## 1. Introduction

Total hip arthroplasty (THA) has emerged as a highly successful surgical intervention that improves the life of patients suffering from hip joint osteoarthritis by alleviating pain and restoring functionality [1,2,3]. Both cemented and uncemented femoral components in THA demonstrated outstanding long-term survival rates exceeding 95% after a decade [4]. Despite this, aseptic loosening is, in general, the leading cause of stem revision [5,6,7,8,9,10]. Aseptic loosening can occur from an inadequate initial fixation [11]. While the insufficient fixation of cementless femoral components arises from an inadequate press fit resulting in micromotion with a subsequent lack of osseointegration, a lack of fixation in cemented stems can result from either a suboptimal cementation technique or component malposition [11,12].

In this context, earlier research findings indicated that distal migration of the stem, commonly referred to as subsidence, serves as a reliable predictive indicator for early aseptic loosening [13,14,15,16,17]. However, different migration thresholds have been published in the past depending on the used measurement method. By using Roentgenstereophotogrammetrie (RSA), Kärrholm et al. reported that the subsidence of cemented stems exceeding 1.2 mm within the initial 2 years after surgery is likely to result in an early implant failure within the subsequent 4–7 years [16]. Next to this, Krismer et al. rated a distal migration of 1.5 mm measured with Einzel-Bild-Roentgen-Analyse–Femoral Component Analysis (EBRA-FCA) after 2 years as a risk factor for early aseptic loosening [13].

EBRA-FCA represents a computerized approach for assessing the distal migration of femoral stems through conventional anterior–posterior (AP) pelvic radiographs, eliminating the need for supplementary tools like ball markers during exposure. In comparison to roentgen stereophotogrammetric analysis (RSA), EBRA-FCA has demonstrated a reliable measurement accuracy in detecting axial migration exceeding 1 mm, coupled with excellent interobserver reliability [13,18].

In addition to radiological criteria, the clinical outcome plays a decisive role in the assessment of hip prosthesis. For this purpose, various methods are currently available encompassing patient-based, functional-performance-related, and surgeon-based evaluations [19]. Among surgeon-based outcome measures, range of motion has been described to correlate with stair-climbing ability, tying shoes, as well as patients’ opinion of their functional status [20], and it was found to be a sensitive indicator of hip outcome after THA [21].

The investigated stem is the cemented Lubinus SP2 by Link (Waldemar Link, Hamburg, Germany), which has an anatomic design with an S-shaped curvature in the sagittal plane [22].

This study aimed to examine the clinical outcomes and migration patterns of the cemented SP2 stem using EBRA-FCA during a median mid-term follow-up of 6.5 years. Additionally, we assessed the potential impact of femoral configuration and body mass index (BMI).

## 2. Materials and Methods

This study was approved by the local ethics committee. In the present retrospective study, all subsequent patients who received a cemented Lubinus SP2 stem at the Department of Orthopaedics and Traumatology of the Medical University of Innsbruck between 2003 and 2019 were reviewed. During this period, a total of 244 cemented Lubinus SP2 stems were implanted.

The Lubinus SP2 stem is a Cobalt–Chrome–Molybdenum-tapered, anatomically designed prosthesis featuring a collar, originally introduced in 1982 [22]. Presently, this prosthesis is provided in three standard lengths (130, 150, and 170 mm), encompassing three Center–Collum–Diaphysis (CCD) angles (117°, 126°, and 135°), and available in seven distinct stem widths [22]. Additionally, the stem offers a standard and an extra-long neck, along with up to four head–neck lengths to precisely accommodate lateralization and leg-length requirements [22].

The prosthetic design, characterized by its anatomical shape, facilitates the formation of a consistent cement mantle around the stem within the medullary canal [22]. This anatomical feature contributes to a more natural stress distribution by mitigating pinpoint stress at the bone/implant interface [22,23].

This investigation involved a thorough examination of medical histories, with a focus on gathering sociodemographic information, details about the surgical approach employed, BMI, cut-to-suture time, and the preoperative diagnosis indicating total hip arthroplasty (THA). Additionally, the femoral configuration was systematically categorized using preoperative X-ray images, following the classification scheme proposed by Dorr, which includes type A (“champagne flute”), type B (“normal”), and type C (“stovepipe”) [24]. Furthermore, the range of motion was systematically documented preoperatively and up to one year after surgery by the surgeons in our department, using a goniometer as part of the clinical examination protocol.

**Figure 1:**
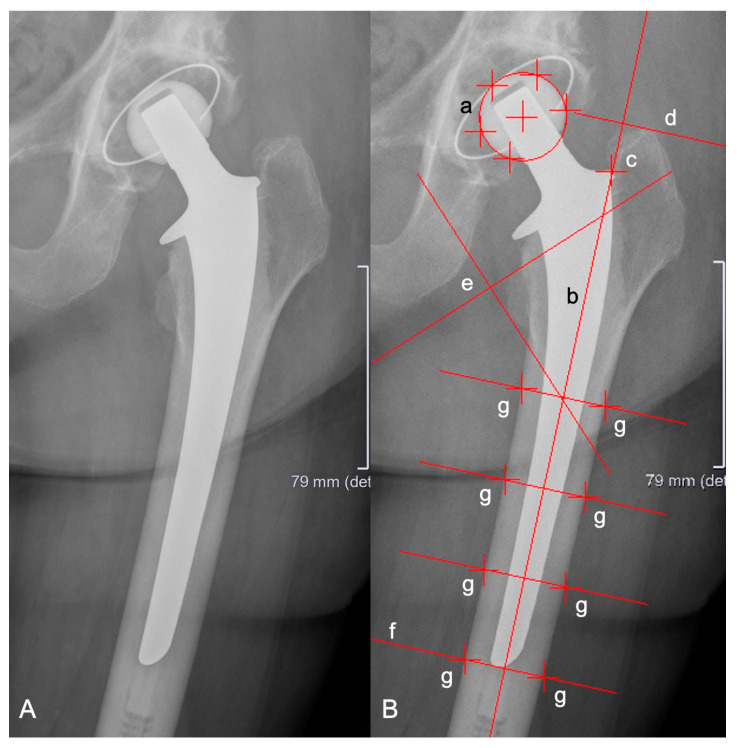
Anterior-to-posterior X-rays showing a cemented SP2 stem (**A**) and with EBRA-FCA references (**B**): (a) head points, (b) stem axis, (c) stem shoulder, (d) major trochanter line, (e) minor trochanter lines, (f) tip-of-stem line, and (g) points at femoral bone contour.

Axial migration of the stem, prosthetic stability, as well as stem tilting were retrospectively evaluated with EBRA-FCA (University of Innsbruck, Austria) by using plain X-ray images [13,18]. A total of 19 reference points were determined, distributed across the femoral head (n = 3–7), the stem (n = 2), the femoral cortex (n = 8), and singularly at the major and minor trochanter [18]. The EBRA-FCA software employs a comparability algorithm to exclude unsuitable radiographs. The program measures three distances between bony and prosthetic landmarks (the distance between the center of the femoral head and the stem shoulder, the center of the femoral head and the stem axis, and the stem shoulder and the tip of the stem) and compares these distances between the pairs of radiographs. If the distance exceeds the comparability limit of 3 mm, the image is excluded due to significant positioning artifacts. Figure 1 depicts the X-ray image of a SP2 stem, including EBRA-FCA references [18].

In the Department of Orthopaedics and Traumatology at the Medical University of Innsbruck, a standardized radiographic follow-up protocol is routinely implemented for patients, comprising assessments prior to discharge, at 6 weeks postsurgery, 12 months postoperative, and subsequently at intervals of 1 to 2 years. Additional radiographs are conducted in cases where patients report any complaints related to THA. All radiographic imaging is uniformly performed at our Department of Radiology using the anterior–posterior (AP) projection, with patients assuming an upright position under full weight-bearing conditions. All X-ray images were stored on a Picture Archiving and Communication System (PACS). The inclusion criteria for this investigation necessitated a minimum of three radiographs per patient and a minimum radiological follow-up period of 2 years.

A migration analysis was conducted using EBRA-FCA by an impartial investigator who was not directly involved in the surgical procedures or postoperative care of the patients.

### Statistics

Descriptive statistics (the mean, median, range, and standard deviation) were obtained using Excel (Microsoft Excel 2016, Redmond, WA, USA). All calculations for comparative statistics were carried out using Graph Pad Prism (Version 10.0, GraphPad Software, Inc., La Jolla, CA, USA). The Kolmogorov–Smirnov Test was used to assess the normal distribution of the datasets. As in most of the cases, the data were not normally distributed, the Kruskal–Wallis Test with Dunn’s correction for multiple comparisons was used for the evaluation of the EBRA-FCA measurements at different time steps. The Kruskal–Wallis Test with Dunn’s correction for multiple comparisons was also used for the investigation of patients with different femoral configurations (Dorr type). For assessments of the EBRA measurements for the comparison of patients with different BMIs (BMI  ≤  25 kg/m^2^ and overweight BMI > 25.0 kg/m^2^), the Mann–Whitney U Test was used. The range of motion pre- and postoperatively was analyzed by using the Wilcoxon matched-pairs signed-rank test. A *p*-value of 0.05 was considered to be statistically significant.

## 3. Results

A total of 61 patients who underwent THA surgery and fulfilled the inclusion criteria as well as provided informed consent were enrolled in this study (female: 48, male: 13, ratio 79:21). The mean age of the patients was 76 years (range 30–93), with a median follow-up duration of 6.5 years (range 2–16). Among the patient cohort, 77.0% underwent THA surgery for primary osteoarthritis (OA). A predominant proportion of patients (67.2%) underwent surgical interventions in a lateral position utilizing a transgluteal lateral approach, whereas the remaining cohort (32.8%) underwent procedures in a supine position employing a direct anterior approach. The mean cut-to-suture time was 105 (range 33–282) min. Additional details on the demographic data of the patients as well as the surgical procedure are given in Table 1, while details on the implanted stems can be found in Table 2.

EBRA-FCA was conducted at the final follow-up for 55 out of the 61 stems, utilizing an EBRA-FCA-prescribed comparability limit of 3.0 mm with a 95% confidence interval. On average, 5.3 X-rays (range 3–11) were subjected to analysis for each implant. Six patients were excluded from the EBRA-FCA migration analysis due to issues related to the comparability algorithm. A comprehensive set of X-ray images at each designated time point (e.g., 6 months, 12 months, etc.) was not available for the majority of patients. Owing to the drop out of patients during the follow-up period, varying case numbers were considered in the analysis of migration behavior over time.

EBRA-FCA demonstrated a median migration of 0.2 mm (range 0.0–2.6) at 6 months, 0.5 mm (range 0.0–2.4) at 12 months, 0.7 mm (range 0.0–4.2) at 24 months, and 0.7 mm (range 0.1–6.5) at the last follow-up (median 78 months). The magnitude of subsidence at 6 months and the last follow-up showed a statistically significant difference (*p* < 0.0001). In all other comparisons, no statistical significance could be found for implant subsidence.

**Figure 2:**
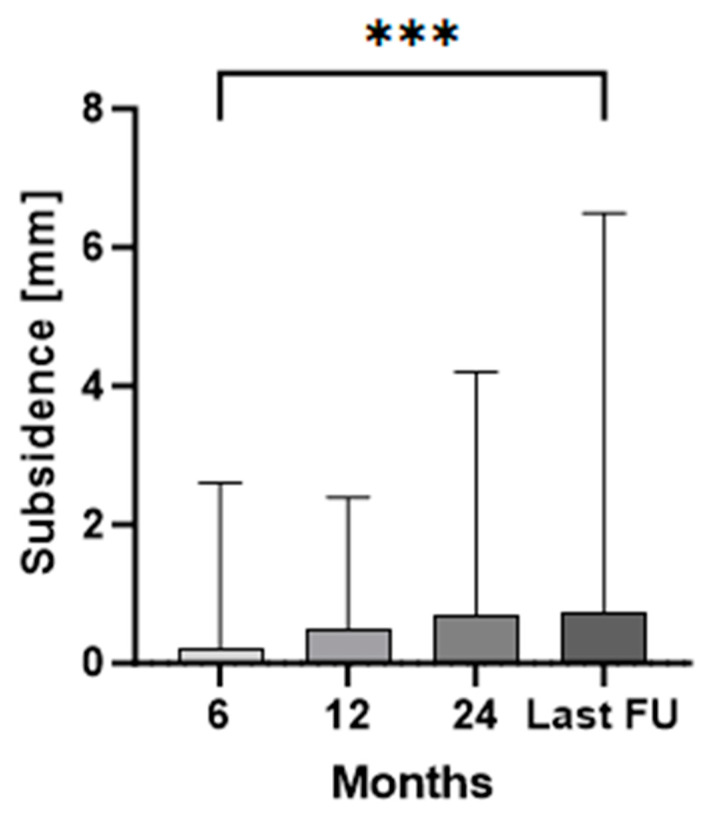
Median and interquartile range (bars) of total stem subsidence for the clinical follow-up. The three asterisks (***) indicate the statistical significance level of *p* < 0.001 between 6 months and last follow-up (FU).

Mean monthly axial implant migration was 0.03 mm/month within the first 6 months, 0.05 mm/month between 6 and 12 months, 0.02 mm/month between 12 and 24 months, and no further migration until the last follow-up. Thus, the main axial subsidence was observed predominantly within the period between six and twelve months postsurgery (Figure 2).

**Figure 3:**
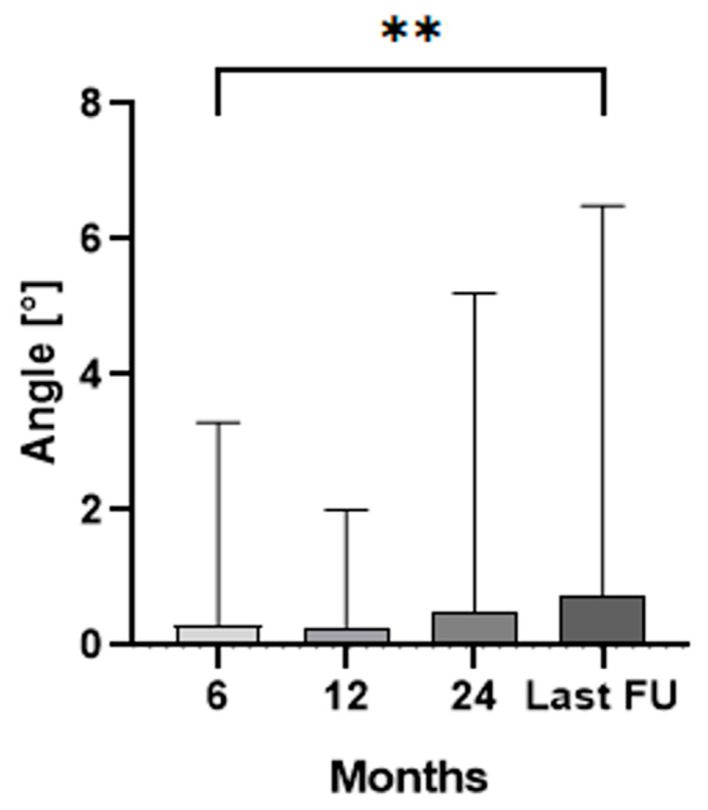
Median and interquartile range (bars) of the angle between stem and femur axis for the clinical follow-up. The two asterisks (**) indicate the statistical significance level of *p* < 0.01 between 6 months and last follow-up (FU).

Additionally, the median angle between the stem and femoral axis measured 0.3° (range 0.0–3.3°) at 6 months, 0.3° (range 0.0–2.0°) at 12 months, 0.5° (range 0.0–5.2°) at 24 months, and 0.8° (range 0.1–17.3°) at the last follow-up (Figure 3). A statistically significant higher angle between the stem and femoral axis could be found between X-rays taken after 6 months and the last follow-up (*p* = 0.007). No statistically significant difference could be found for the angle between the stem and femoral axis for the other intervals (*p* > 0.06).

Patients were split into three groups according to the Dorr classification. At 6 months follow-up, patients classified with Dorr type A showed a statistically significant (*p* = 0.016) higher migration (median subsidence 0.4 mm, range 0.1–2.6) when compared to patients classified with Dorr type B (median subsidence 0.1 mm, range 0.0–1.0). At 6 months of follow-up, no other statistically significant differences could be found for Dorr type C (median subsidence 0.3 mm, range 0.0–1.6, *p* > 0.053). After 6 months of follow-up, no statistically significant difference could be observed between the three Dorr groups. No statistically significant difference could be found between the three Dorr groups when considering the angle variation.

The patients were also categorized into two groups based on their BMI: normal (BMI ≤ 25 kg/m^2^) and overweight (BMI > 25.0 kg/m^2^). There was no statistically significant difference between the two groups in terms of subsidence and angle during the follow-up period (*p* > 0.05).

The pre- and postoperative comparison of the range of motion revealed statistically significant mean improvements: flexion increased by 10° (*p* = 0.0088), internal rotation by 10° (*p* = 0.0002), external rotation by 8° (*p* = 0.0011), adduction by 10° (*p* = 0.0011), and abduction by 7° (*p* < 0.0001). However, no statistically significant difference was observed in the range of motion for extension (*p* = 0.5000) between the pre- and postoperative assessments.

## 4. Discussion

In this study, the migration pattern of a cemented, tapered, anatomically designed prosthesis featuring a collar was analyzed at a median mid-term follow-up of 6.5 years. Additionally, the impact of patient-related attributes on subsidence was assessed. To the authors’ knowledge, this study is the first migration analysis for this prosthesis using the EBRA-FCA method. The most important finding of this study was a median migration of the Lubinus SP 2 stem (Waldemar Link, Hamburg, Germany) of 0.7 mm at both 24- and 78-month follow-up. Although BMI did not demonstrate a statistically significant effect on subsidence, there was a significant increase in distal stem migration observed at 6 months in Dorr type A compared to those classified as Dorr type B.

Failures of implants intended for the proximal femur, whether used in osteosynthesis or hip arthroplasty, can have significant implications for patient outcomes, often necessitating complex revision surgeries [25]. To mitigate the risk of failure, it is essential to know the most common causes and understand the mechanisms of failure for the implant used. In osteosynthesis, implants like gamma nails and dynamic hip screws are often used for the stabilization of fractures in the proximal femur [26,27]. The most frequently observed complication with both implants is the cut-out of the lag screw, which is due to poor positioning in the femoral head [28,29]. In total hip arthroplasty, femoral components vary in design and fixation technique, with common causes for revision including aseptic loosening, infection, and periprosthetic fracture [30].

In cemented stems, aseptic loosening poses a challenge and represents a leading cause for stem revision, with early subsidence being acknowledged as a predisposing risk factor [13,31,32]. With a specificity of 100% and a sensitivity of 78% in identifying distal stem migration exceeding 1 mm in comparison to RSA, EBRA-FCA is considered appropriate for detecting and quantifying subsidence in THA [18]. Although RSA is recognized as the benchmark for migration measurements, its reliance on the implantation of tantalum marker balls limits its applicability to prospective study designs exclusively [33]. Notably, EBRA-FCA offers the advantage of being a non-invasive technique, making it feasible for the retrospective study design in our investigation.

In the past, Kärrholm et al. reported a revision rate for the cemented Lubinus SP 1 stem of over 50% when the subsidence measured by RSA exceeded 1.2 mm within the first 2 years postoperatively, rising to over 95% when distal migration surpassed 2.6 mm [16]. Using EBRA-FCA, Krismer et al. were able to identify a threshold of 1.5 mm within the first two years, where revision due to aseptic loosening can be predicted with a sensitivity of 69% and a specificity of 80% [13]. However, it should be noted that out of a total of 240 investigated implants, only 60 were cemented (MEM, Protek Ltd., Bern, Switzerland), which led to an inhomogeneous group in Krismer et al. [13].

Various implants were part of migration measurements using these threshold values. In their RSA study, Nieuwenhuijse et al. reported a mean distal migration of the cemented Exeter stem (Stryker, Kalamazoo, MI, USA) of 2.13 mm after a ten-year follow-up, whereas the stem continued to migrate during the whole first decade after implantation [17]. The continuous migration of the Exeter stem was confirmed by Murray et al., with a mean observed migration at two years of 0.7 mm and 1.3 mm at 10 years, which is less than what was reported in the aforementioned study [17,34]. Clement et al. documented a subsidence of 1.2 mm in the Exeter stem two years after surgery by using EBRA-FCA for migration measurement [35]. As Nieuwenhuijse et al. had no stem revision in their cohort, the authors suggested that absolute stability is not a prerequisite for sufficient long-term follow-up if the stem design is aligned with migration compatibility [17,36]. Moreover, Murray et al. described the migration of the Exeter stem as a combination of subsidence and internal rotation with posterior head migration, whereby subsidence occurred within the cement mantle [34]. Based on these findings, the authors concluded that a sustained compressive force is generated at the cement and bone–cement interface, maintaining a secure fixation over the long term [34]. In comparison, Sesselmann et al. examined the migration behavior of the Lubinus SP 2 stem by using RSA [37]. Herein, the stem exhibited a lesser distal migration of 0.03 mm at 2 years and of 0.04 mm at 10 years [37]. The maximum subsidence occurred between 6 and 12 months postoperatively [37]. In our study, we were able to confirm the observation reported by Sesselmann et al. regarding the timing of the greatest migration. However, we found a higher median distal migration of 0.7 mm after 2 years and at the last follow-up examination using EBRA-FCA. Nevertheless, these results are clearly below the reported thresholds of Kärrholm and Krismer [13,16].

The difference in the migration behavior of the mentioned stems is explained by their stem designs [38]. The Exeter stem operates as a force-closed stem, which is characterized by its collarless, double-tapered polished configuration [17,34]. This structural arrangement is known to promote distal migration within the cement mantle [17,34]. In contrast, the SP 2 stem is a shape-closed stem characterized by a collar that facilitates the physiological force reintroduction into the femur, while resisting rotational forces due to its s-shaped design resulting in almost no further migration one year postoperatively [39].

Different femoral configurations (“champagne flute”, “normal”, and “stovepipe”) should be considered when selecting the stem type [23,24]. Femora classified as Dorr type A with thick and pronounced cortices forming a narrow diaphyseal canal and a “champagne flute” of the proximal femur are of particular interest [39,40]. Jørgensen et al. found significantly higher subsidence in Exeter standard stems compared with the Exeter short stem in Dorr type A femora within the first 3 months (1.59 mm vs. 0.89 mm, *p* < 0.001) [40]. Afterward, both stems showed a similar migration pattern of continued subsidence and retroversion [40]. Prins et al. conducted a study revealing excellent long-term results with the relatively short 130 mm SP 2 stem. They highlighted potential benefits such as the preservation of bone distal to the stem and the improved proximal filling, resulting in a more even cement distribution [39]. This might be particularly advantageous in Dorr type A femurs [39]. However, the authors did not measure stem subsidence [39]. In our research, patients classified as Dorr type A exhibited significantly (*p* = 0.016) higher migration rates after 6 months compared to those categorized as Dorr type B (0.4 mm vs. 0.1 mm). Subsequently, no further difference in migration between the groups was found at the other time points. It is important to mention that our study group did not include 130 mm Lubinus SP II stems.

The influence of other demographic factors like weight or BMI on stem subsidence yielded different results for cemented and cementless stems [41,42,43]. While in the study by Dammerer et al. the subsidence within the first 12 months for a cementless Accolade II stem (Stryker, Kalamazoo, MI, USA) was increased in obese patients (BMI ≥ 30 kg/m^2^), Stihsen et al. were only able to demonstrate an influence of increased body weight, but not BMI, on migration for the cementless Vision 2000 stem (Depuy, Warsaw, IN, USA) [41,42]. When having a look at cemented stems, Onsten et al. demonstrated, in an RSA study, that body weight had no influence on distal stem migration [43]. In the present study, a BMI > 25 kg/m^2^ was not found to yield a statistically significant increase in subsidence.

This study has some limitations that must be mentioned. First, this study has a retrospective design, and thus some of the patients had to be excluded from the cohort, which may make the study more susceptible to selection bias. Second, a control group was missing. Third, variations in the number of X-rays taken for each hip during follow-up, along with the smoothing function of EBRA-FCA, might have impacted the migration results. Fourth, no particular hip score was accessible for assessing clinical outcomes.

## 5. Conclusions

In summary, the subsidence of the Lubinus SP 2 stem measured with EBRA-FCA was below the known thresholds and in line with the existing literature, suggesting excellent long-term results. The subsidence in Dorr type A femora was significantly increased within the first 6 months but subsequently showed no further statistical difference to Dorr type B femora. BMI had no influence on subsidence.

## Figures and Tables

**Table 1:** Patient demographics of the study group. Range is given in brackets.

Number of Patients	Female	48
	Male	13
Mean age (years)		76 (30–93)
Mean BMI (kg/m^2^)		26 (19–34)
Surgical site	Left	27
	Right	34
Median radiological follow-up (years)		6.5 (2–16)
Preoperative diagnosis	Primary osteoarthritis	47
	Femoral neck fracture	7
	Avascular necrosis of femoral head	5
	Others	2
Surgical approach	Direct anterior approach	20
	Transgluteal lateral approach	41
Surgical position	Supine	20
	Lateral	41
Mean cut-to-suture time (min)		105 (33–282)

**Table 2:** Details on Dorr classification and implanted components. Percentage given in brackets.

Dorr Classification	A	15 [24.6]
	B	26 [42.6]
	C	20 [32.8]
Stem length	130	0 [0.0]
	150	55 [90.2]
	170	6 [9.8]
CCD angle	117	0 [0.0]
	126	26 [42.6]
	135	35 [57.4]
Head diameter (mm)	28	33 [54.1]
	32	28 [45.9]

## Data Availability

The data presented in this study are available upon request from the corresponding author.

## References

[1] Pivec R., Johnson A.J., Mears S.C., Mont M.A. (2012). Hip Arthroplasty. Lancet.

[2] Li W., Ayers D.C., Lewis C.G., Bowen T.R., Allison J.J., Franklin P.D. (2017). Functional Gain and Pain Relief After Total Joint Replacement According to Obesity Status. J. Bone Jt. Surg. Am..

[3] Okafor L., Chen A.F. (2019). Patient Satisfaction and Total Hip Arthroplasty: A Review. Arthroplasty.

[4] Kärrholm J., Mohaddes M., Odin D., Vinblad J., Rogmark C., Rolfson O. (2018). Swedish Hip Arthroplasty Register Annual Report 2017. https://registercentrum.blob.core.windows.net/sar/r/Swedish-Hip-Arthroplasty-Register-Annual-report-2017-BylrAoP8ro.pdf.

[5] Melvin J.S., Karthikeyan T., Cope R., Fehring T.K. (2014). Early Failures in Total Hip Arthroplasty—A Changing Paradigm. J. Arthroplast..

[6] Johnsen S.P., Sørensen H.T., Lucht U., Søballe K., Overgaard S., Pedersen A.B. (2006). Patient-Related Predictors of Implant Failure after Primary Total Hip Replacement in the Initial, Short- and Long-Terms. A Nationwide Danish Follow-up Study Including 36,984 Patients. J. Bone Jt. Surg. Br..

[7] Ulrich S.D., Seyler T.M., Bennett D., Delanois R.E., Saleh K.J., Thongtrangan I., Kuskowski M., Cheng E.Y., Sharkey P.F., Parvizi J. (2008). Total Hip Arthroplasties: What Are the Reasons for Revision?. Int. Orthop..

[8] Sadoghi P., Liebensteiner M., Agreiter M., Leithner A., Böhler N., Labek G. (2013). Revision Surgery after Total Joint Arthroplasty: A Complication-Based Analysis Using Worldwide Arthroplasty Registers. J. Arthroplast..

[9] Haynes J.A., Stambough J.B., Sassoon A.A., Johnson S.R., Clohisy J.C., Nunley R.M. (2016). Contemporary Surgical Indications and Referral Trends in Revision Total Hip Arthroplasty: A 10-Year Review. J. Arthroplast..

[10] Feng X., Gu J., Zhou Y. (2022). Primary Total Hip Arthroplasty Failure: Aseptic Loosening Remains the Most Common Cause of Revision. Am. J. Transl. Res..

[11] Anil U., Singh V., Schwarzkopf R. (2022). Diagnosis and Detection of Subtle Aseptic Loosening in Total Hip Arthroplasty. J. Arthroplast..

[12] Katzer A., Lœhr J.F. (2003). Early Loosening of Hip Replacements: Causes, Course and Diagnosis. J. Orthopaed Traumatol..

[13] Krismer M., Biedermann R., Stöckl B., Fischer M., Bauer R., Haid C. (1999). The Prediction of Failure of the Stem in THR by Measurement of Early Migration Using EBRA-FCA. J. Bone Jt. Surg Br..

[14] Freeman M.A., Plante-Bordeneuve P. (1994). Early Migration and Late Aseptic Failure of Proximal Femoral Prostheses. J. Bone Jt. Surg. Br..

[15] Kroell A., Beaulé P., Krismer M., Behensky H., Stoeckl B., Biedermann R. (2009). Aseptic Stem Loosening in Primary THA: Migration Analysis of Cemented and Cementless Fixation. Int. Orthop..

[16] Kärrholm J., Borssén B., Löwenhielm G., Snorrason F. (1994). Does Early Micromotion of Femoral Stem Prostheses Matter? 4–7-Year Stereoradiographic Follow-up of 84 Cemented Prostheses. J. Bone Jt. Surg. Br..

[17] Nieuwenhuijse M.J., Valstar E.R., Kaptein B.L., Nelissen R.G.H.H. (2012). The Exeter Femoral Stem Continues to Migrate during Its First Decade after Implantation: 10–12 Years of Follow-up with Radiostereometric Analysis (RSA). Acta Orthop..

[18] Biedermann R., Krismer M., Stöckl B., Mayrhofer P., Ornstein E., Franzén H. (1999). Accuracy of EBRA-FCA in the Measurement of Migration of Femoral Components of Total Hip Replacement. J. Bone Jt. Surg. Br..

[19] Wylde V., Blom A.W. (2009). Assessment of Outcomes after Hip Arthroplasty. HIP Int..

[20] Bryant M.J., Kernohan W.G., Nixon J.R., Mollan R.A. (1993). A Statistical Analysis of Hip Scores. J. Bone Jt. Surg. Br..

[21] Davis K.E., Ritter M.A., Berend M.E., Meding J.B. (2007). The Importance of Range of Motion after Total Hip Arthroplasty. Clin. Orthop. Relat. Res..

[22] Lubinus SP II Anatomically Adapted Cemented Hip System—Surgical Technique 2020. https://www.link-ortho.com/fileadmin/user_upload/Fuer_den_Arzt/Produkte/Downloads/EN/6431_SP_II_OP-Impl-Instr_en_2020-03_001_MAR-02619_1-0.pdf.

[23] Noble P.C., Alexander J.W., Lindahl L.J., Yew D.T., Granberry W.M., Tullos H.S. (1988). The Anatomic Basis of Femoral Component Design. Clin. Orthop. Relat. Res..

[24] Dorr L.D., Faugere M.C., Mackel A.M., Gruen T.A., Bognar B., Malluche H.H. (1993). Structural and Cellular Assessment of Bone Quality of Proximal Femur. Bone.

[25] Müller F., Galler M., Zellner M., Bäuml C., Füchtmeier B. (2017). Total Hip Arthroplasty after Failed Osteosynthesis of Proximal Femoral Fractures: Revision and Mortality of 80 Patients. J. Orthop. Surg..

[26] Valverde J.A., Alonso M.G., Porro J.G., Rueda D., Larrauri P.M., Soler J.J. (1998). Use of the Gamma Nail in the Treatment of Fractures of the Proximal Femur. Clin. Orthop. Relat. Res..

[27] Chang C.-W., Chen Y.-N., Li C.-T., Peng Y.-T., Chang C.-H. (2015). Role of the Compression Screw in the Dynamic Hip–Screw System: A Finite-Element Study. Med. Eng. Phys..

[28] Bojan A.J., Beimel C., Taglang G., Collin D., Ekholm C., Jönsson A. (2013). Critical Factors in Cut-out Complication after Gamma Nail Treatment of Proximal Femoral Fractures. BMC Musculoskelet. Disord..

[29] Hsueh K.-K., Fang C.-K., Chen C.-M., Su Y.-P., Wu H.-F., Chiu F.-Y. (2010). Risk Factors in Cutout of Sliding Hip Screw in Intertrochanteric Fractures: An Evaluation of 937 Patients. Int. Orthop..

[30] Oltean-Dan D., Apostu D., Tomoaia G., Kerekes K., Păiuşan M.G., Bardas C.-A., Benea H.R.C. (2022). Causes of Revision after Total Hip Arthroplasty in an Orthopedics and Traumatology Regional Center. Med. Pharm. Rep..

[31] Cassar-Gheiti A.J., McColgan R., Kelly M., Cassar-Gheiti T.M., Kenny P., Murphy C.G. (2020). Current Concepts and Outcomes in Cemented Femoral Stem Design and Cementation Techniques: The Argument for a New Classification System. EFORT Open Rev..

[32] Hanif M., Arshad N., Habib Y., Shami A.M., Rehman O.U., Rehman M., Reyaz M., Mumtaz H. (2023). Effect of Cementing Technique on Aseptic Stem Loosening in Cemented Primary Total Hip Arthroplasty: A Systematic Review and Meta-Analysis. Ann. Med. Surg..

[33] Selvik G. (1990). Roentgen Stereophotogrammetric Analysis. Acta Radiol..

[34] Murray D.W., Gulati A., Gill H.S. (2013). Ten-Year RSA-Measured Migration of the Exeter Femoral Stem. Bone Jt. J..

[35] Clement N.D., Bardgett M., Merrie K., Furtado S., Bowman R., Langton D.J., Deehan D.J., Holland J. (2019). Cemented Exeter Total Hip Arthroplasty with a 32 Mm Head on Highly Crosslinked Polyethylene: Does Age Influence Functional Outcome, Satisfaction, Activity, Stem Migration, and Periprosthetic Bone Mineral Density?. Bone Jt. Res..

[36] Mancino F., Tornberg H., Jones C.W., Bucher T.A., Malahias M.-A. (2023). The Exeter Cemented Stem Provides Outstanding Long-Term Fixation and Bone Load at 15 Years Follow-up: A Systematic Review and Meta-Analysis. J. Orthop. Surg..

[37] Sesselmann S., Hong Y., Schlemmer F., Wiendieck K., Söder S., Hussnaetter I., Müller L.A., Forst R., Wierer T. (2017). Migration Measurement of the Cemented Lubinus SP II Hip Stem—A 10-Year Follow-up Using Radiostereometric Analysis. Biomed. Tech..

[38] van der Voort P., Pijls B.G., Nieuwenhuijse M.J., Jasper J., Fiocco M., Plevier J.W.M., Middeldorp S., Valstar E.R., Nelissen R.G.H.H. (2015). Early Subsidence of Shape-Closed Hip Arthroplasty Stems Is Associated with Late Revision. A Systematic Review and Meta-Analysis of 24 RSA Studies and 56 Survival Studies. Acta Orthop..

[39] Prins W., Meijer R., Kollen B.J., Verheyen C.C., Ettema H.B. (2014). Excellent Results with the Cemented Lubinus SP II 130-Mm Femoral Stem at 10 Years of Follow-up: 932 Hips Followed for 5–15 Years. Acta Orthop..

[40] Jørgensen P.B., Jakobsen S.S., Vainorius D., Homilius M., Hansen T.B., Stilling M. (2023). Less Early Subsidence of Cemented Exeter Short Stems Compared with Cemented Exeter Standard Stems in Dorr Type A Femurs. Bone Jt. Open.

[41] Dammerer D., Blum P., Putzer D., Krappinger D., Liebensteiner M.C., Nogler M., Thaler M. (2022). Subsidence of a Metaphyseal-Anchored Press-Fit Stem after 4-Year Follow-up: An EBRA-FCA Analysis. Arch. Orthop. Trauma. Surg..

[42] Stihsen C., Radl R., Keshmiri A., Rehak P., Windhager R. (2012). Subsidence of a Cementless Femoral Component Influenced by Body Weight and Body Mass Index. Int. Orthop..

[43] Onsten I., Akesson K., Besjakov J., Obrant K.J. (1995). Migration of the Charnley Stem in Rheumatoid Arthritis and Osteoarthritis. A Roentgen Stereophotogrammetric Study. J. Bone Jt. Surg. Br..

